# Delivery of large molecular protein using flat and short microneedles prepared using focused ion beam (FIB) as a skin ablation tool

**DOI:** 10.1007/s13346-015-0252-0

**Published:** 2015-07-31

**Authors:** Karmen Cheung, Geoff West, Diganta Bhusan Das

**Affiliations:** Department of Chemical Engineering, Loughborough University, Loughborough, LE11 3TU UK; Department of Materials, Loughborough University, Loughborough, LE11 3TU UK

**Keywords:** Flat microneedles, Skin ablation, Focused ion beam (FIB), Scanning electron microscope, Infinite focus microscopy

## Abstract

Many studies have been reported in the literature on the effects of various geometries and lengths of microneedles (MNs) on transdermal drug delivery using a variety of drug molecules. In particular, sharp-tipped MNs have been used to disrupt the top layer of the skin, namely, *stratum corneum* (SC). It has also been shown that short- and flat-tipped MNs can pierce the SC and they have the potential to increase drug permeability. However, there is little work that explores MNs as a skin ablative tool with a view to increasing skin permeability. To address this point, well-defined small patterns (size of individual pattern 10–20 μm) on the tip of flat MN (tip radius of individual MN ∼250 μm) were created and their effects evaluated on the permeability of bovine serum albumin (BSA), which is chosen as a model drug of high molecular weight. The patterns on the tip of flat MN act as rough surfaces (e.g. like sand paper) which when applied on the surface of the skin ablate the SC layer. Focused ion beam (FIB) has been used as the fabrication technique for the MNs. The permeability data are then compared with the other data for flat- and sharp-tipped MN. The permeability data from passive diffusion experiments are used as the reference case. The exact number of MNs or patterns in the flat and patterned MN patches is not considered as important as they have not been designed to pierce the skin. However, this is an important consideration in the case of sharp MNs as they pierce and create cavities in the skin. It is found that the delivery of BSA with the fabricated flat and patterned MNs gave similar but somewhat lower drug permeation profile in comparison to the sharp MNs. Passive diffusion showed no permeation, as would be expected due to the large size of the chosen molecule.

## Introduction

Microneedles (MNs) are a transdermal drug delivery system that combines the technology of transdermal patches and hypodermic needles. There are two key types of MNs, namely, solid and hollow MNs [1, 2]. The materials that have been used to fabricate the MNs range from metals [3], glass [4], silicon [5], biodegradable polymers [6, 7] and silk fibroin [8]. Ideally, these materials would be pharmacologically inert, non-toxic and compatible with pharmaceutical ingredients, etc. [9]. The metals traditionally used for MN fabrication consist of stainless steel, nickel coated in gold, titanium, platinum and palladium [10, 11].

MNs have been used to deliver several high molecular weight drugs. For example, bovine serum albumin (BSA) has been used as a model drug in several journal papers [12–14] to characterise the role of various MN geometries on drug permeation in the skin. MNs perform by disrupting the skin and thereby increasing drug permeability. MNs are scarcely less than 150 μm in length, but a study conducted by Wei-Ze et al. [5] explored the fabrication of super short MNs with a length of 70–80 μm. These authors compared the use of sharp-tipped super-short MNs against blunt super-short MNs and longer sharp needles of 1500 μm. Their results concluded that both sharp and blunt super-short MNs are capable of successfully delivering the Alzheimer’s drug galanthamine [5].

Passive diffusion of molecules can typically occur with molecules that are less than 500 Da [15]. Therefore, in order to allow permeation of larger molecules such as BSA (approximately 66.5 kDa), the rate-limiting barrier of the skin, i.e. the stratum corneum layer, needs to be disrupted in some form. For this purpose, various skin ablation techniques involving micro- or nano-derma abrasion have been attempted [16–18]. However, there is little discussion on how these methods compare with MNs. Furthermore, there is little or no work that explores MNs as a tool for ablating the skin surface.

In addressing these points, this short communication will note the effect of flat-tipped MN and a well-defined patterned MN that ablates the skin to compare the permeation of BSA as a model drug. These permeation data will also be compared to the data from a sharp-tipped MN. Focused ion beam (FIB) is used as the manufacturing technique that can facilitate the fabrication of a well-defined geometry on the tip surface of a flat MN, which is used to ablate the skin. This is in contrast to the MNs used in the study by Wei-Ze et al. [5], where the MNs were used to pierce the skin. An attempt will be made to evaluate if having a rough surface on the tip of the flat MN tip will have any effect on the drug permeability.

There are multiple technological advances in transdermal drug delivery research that are currently being undertaken. For example, various combinational methods where microneedles have been combined with ultrasound and iontophoresis have been tried [13, 19]. Another example is the use of laser-engineered dissolving MN arrays for the delivery of vaccinations [20].

FIB has been used in this work as it is a technique that can allow a micron-sized shape to be prepared on the tip of the flat MN with good accuracy. This is a novel method to ascertain if applying a pattern on to the tip of a flat MN surface has an effect on permeability. The exact number of patterns on the tip of the well-defined patterned MN is not considered as the aim of this paper is to establish whether a physical ablation technique has an effect on permeability, compared to physically piercing skin.

## Materials and methods

### Materials

BSA and methylene blue were purchased from Sigma-Aldrich (Gillingham, Dorset, UK). A reverse phase high-performance liquid chromatography (RP-HPLC) instrument (Agilent Series 1100, Cheadle Cheshire, UK) was used to determine BSA concentration. Two reagents, acetonitrile and trifluoroacetic acid (TFA), were used as the mobile phases for the HPLC analyses. They were obtained from Fisher Scientific UK Ltd (Loughborough, UK). A Jupiter C4 300A HPLC column (length 150 mm, internal diameter 4.6 mm) column equipped with a security guard column fitted with a Widepore C4 (4 × 3.0 mm) cartridge (Phenomenex, Inc., Macclesfield, UK) was used to quantify the samples containing BSA. A manual Franz diffusion cell (FDC) (Logan Instruments Corporation, New Jersey, USA) was used to conduct the permeation studies with and without MNs. Deionised water purified using a Millipore Elix System (Billerica, MA, USA) was used for all FDC experiments. Fresh porcine full thickness skin was purchased from Welsh School of Pharmacy, Cardiff University, Cardiff (UK) in pre-cut 2.5-cm^2^ sections. The porcine skin samples were prepared by first removing the porcine ear from the main body of the animal. The hairs were then carefully shaved off the ear using electric clippers to ensure the surface of the skin was not damaged. The skin was then removed off the ear cartilage with a scalpel and cut into 2.5-cm^2^ sections. Samples were shipped via special delivery in insulated packaging on the same day of excision. The skin samples are then stored in the freezer at −22 °C. Before the permeation experiments, samples were placed in a beaker of deionised water to thaw for 1 h and then patted dry with laboratory tissue. Commercially available microneedle patch with 1500-μm-long MN was purchased from AdminPatch (Sunnyvale, CA, USA) and used to pre-treat the porcine skin. It was shown by Cheung et al. [21] that the length of this MN does not completely create a cavity in the skin, which has the same length as the MN. This is due to the viscoelasticity of the skin which causes smaller holes [13]. This patch has been proved to help passage of large molecules through the skin [13, 21]. This MN patch has been used in previous work to characterise the BSA release in FDC experiments and therefore would be good to relate the flat and FIB fabricated MNs. There are 31 individual needles on this MN patch, and each is 1400 μm in length. The main characteristics of the MNs have been reported previously by Cheung et al. and are not repeated here [21].

### BSA release measurements using Franz diffusion cell

A Franz diffusion cell apparatus was used to measure the BSA permeability in skin [18]. One thousand microgrammes per millilitre of BSA containing solution was placed into the donor chamber and samples extracted from the receiving compartment at time points of 15 min, 30 min and 1, 1.5, 2, 3, 4 and 5 h. The procedures for conducting the permeation experiments are similar to the work conducted by Han and Das [13], and therefore, they are not discussed in this paper in detail.

### MN insertion into porcine skin

The porcine skin in this study was not stretched when a force was applied which was conducted in a similar manner to MN insertion by Cheung et al. [21]. An in-house force device was used to insert the MN. The method for this is outlined by Cheung et al. [21]. After an MN array was placed onto the porcine skin, it was removed and placed onto the receptor chamber of a diffusion cell. The properties of the purchased AdminPatch® 1500 MN and skin are also shown in Cheung et al. [21].

### Method of analysing BSA concentration using HPLC

The concentration of the sample from the FDC experiment was analysed by RP-HPLC. The diode array detector (DAD) was set at 232 nm. A spectrophotometer (SHIMADZU UV mini 1240) was used to determine the specific wavelength when the light absorbance was at the maximum. A gradient method was adopted with eluent A: 0.1 % TFA in water and B: 0.08 % TFA in acetonitrile, with a mobile phase ratio of A: B, 95:05 to 20:80. The sample size of each injection was 10 μL. The temperature was set to 24 °C. The flow rate was set to 1 ml/min. A complete RP-HPLC run took approximately 20 min with a down time of 2 min between each run. An external standard approach was used for the standardisation for the analysis of the component sample BSA. External standard samples were prepared using pure chemicals of BSA purchased from Sigma-Aldrich and dissolved in deionised water, purified by Millipore Milli-Q Plus 185. Standards of concentration ranging 10–100 μg/ml BSA were prepared and analysed by HPLC to obtain the absorbance.

### Preparation of flat and patterned MNs

As mentioned earlier, this note will compare the use of flat MNs and FIB patterned MNs to create a sand paper-like texture and bought MNs (AdminPatch® 1500) to investigate the amount of BSA permeation. Two hundred fifty-micrometre-diameter, 250-μm-long flat MNs were fabricated to look at the effects of flat MNs on the concentration of bovine serum albumin (BSA) compared to a long sharp MN.

**Fig. 1:**
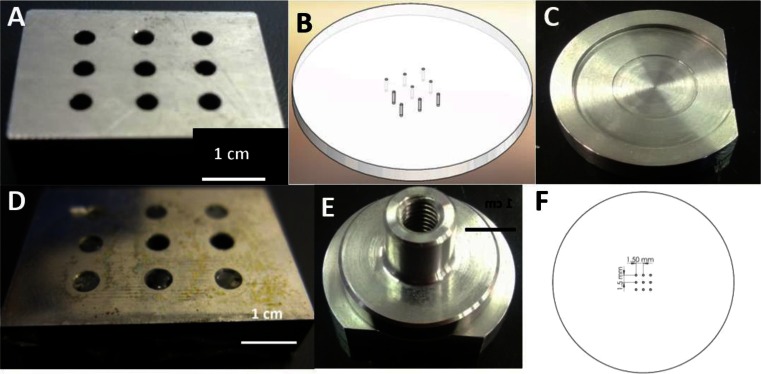
**a** Stainless steel mounting block, **b** microneedle backing plate, **c** stainless microneedle mould mount, **d** ground surface of 250 μm stainless steel wire mounted in stainless steel block held by modelling wax, **e** MN mould and mount with the MN contained inside and **f** MN geometry pitch 1.5 mm

A stainless steel mounting block (H × L × W: 5 mm × 25 mm × 17 mm) with nine 2-mm-diameter drilled holes (Fig. 1a) was machined to mount 250-μm-diameter stainless steel wires upright. The steel wires were purchased from Goodfellow Cambridge Limited (Huntingdon, UK). A translucent Perspex sheet of 1 mm thickness was used to make the MN base unit and cut using a milling machine. This had a 3 × 3 array of nine holes with 0.25 mm diameter drilled with a 1.5-mm spacing (as shown in Fig. 1b). A stainless steel MN mould with a 0.25-mm depth well drilled into it allowed the wires to be mounted at the same height (0.25 mm) (Fig. 1c). To prepare flat MNs, the stainless steel wires were thread into the stainless steel block (Fig. 1a). Hot modelling wax was then placed inside the hole to hold the wire in place. The top surface of the block was then ground for approximately 5 min for each grade of silicon carbide paper (dampened with water), using coarse grit sizes, first, 220, 1200 and then 2400, manufactured by Struers. This was the method to create the flat MN. This same starting point was used to create the FIB patterned MN. The patterning was performed in an FEI Nov. 600 Nova Nanolab dual beam Focused Ion Beam/Field Emission Gun Scanning Electron Microscope (FIB/FEG-SEM). Simple pattern templates were produced by designing a grid pattern that contained 52 15 μm × 15 μm square patterns in Photoshop and were input directly into the patterning engine of the dual beam as a bitmap image. Patterning was performed using a 30-kV Ga^+^ beam and a current of 20 nA, and the cutting progress was monitored using the SEM in real time.

Flat MNs were assembled by removing the ground wires from the mounted block and threading them through the 0.25-mm-diameter holes perspex mounting disc (Fig. 1b) (ensuring the ground end was not threaded through). Once all the wires were threaded, the MN disc was then placed onto the PTFE MN mould with the ground end of the wires pushed gently into the mould to ensure all the needles were at the same length (Fig. 1c). The wires were then glued in place with araldite and left to dry in the mould. A MN holder was then placed on top to conceal the excess wires (Fig. 1e). This was also the mounting piece for allowing a specific force to be applied to porcine skin. Figure 1e depicts the MN mould and mount with the MN contained inside. The patterned MN was mounted in a similar manner. The MN would be a nine-array MN with a pitch of 1.5 mm. The MNs made using FIB technique were fabricated to ascertain if the flat MNs that were patterned allowed more drug to permeate through porcine skin compared to the flat MNs that were produced.

### Characterisation of Microneedles

Surface morphology characterisation was conducted using optical microscopy, SEM and infinite focus 3D microscopy (Graz, Austria). These analysis techniques were used to determine if the surface of the MN was uniform. A Canon (Surry, UK) microscope was used to initially visualise if the top surface of the needles were ground flat. SEM images were used to image pre- and post-ion beam milling of the material. 3D microscopy images were taken using the instrument Infinite Focus by Alicona (Kent, UK).

## Results and discussion

### Characterisation of MNs

**Fig. 2:**
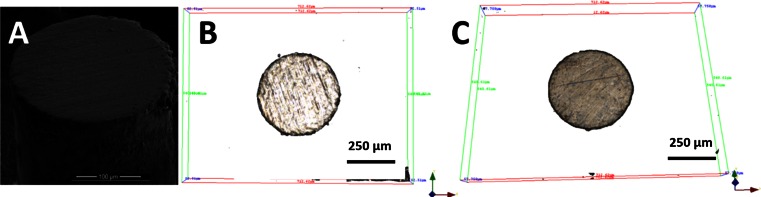
**a** Single SEM image and **b**, **c** single 3D microscopy images of 250 μm stainless steel wires ground using three grades of silicon carbide paper to make flat short MNs

The flat MNs were analysed using microscopy to visualise their initial profiles in order to ascertain if further grinding was required. Once the needles were sufficiently flat, they were then characterised using 3D microscopy to characterise the flat tip profile, as shown in Fig. 2a–c. Using various grades of silicon carbide paper was an effective technique to obtain flat MN structures. It can be seen that there is no burring on the sides of the needles, even if the method used excessive force to grind the needles. This is because the modelling wax used to hold to wired prevented any movement. The 3D microscopy images as shown in Fig. 2b, c show further that the silicon carbide paper produced flat profile MNs.

**Fig. 3:**
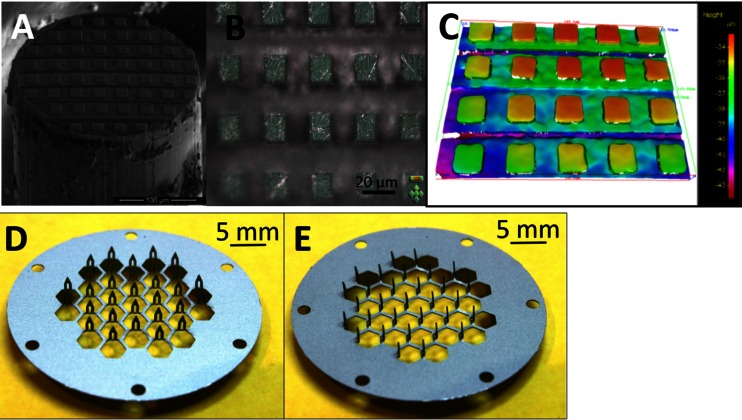
**a** SEM image of a completed FIB needle and **b**, **c** single 3D microscopy images of 250 μm stainless steel wires ground using three grades of silicon carbide paper to make flat MN prior to micro-patterning with FIB. **c** A contoured coloured image of the FIB microneedle. **d** Front view image of the AdminPatch array (1500 μm long); **e** side image of the AdminPatch Array (1500 μm long)

The patterned MNs were initially produced and characterised in an identical manner to the flat MNs. These images are depicted in Fig. 3a–c. The flat MN was placed into a FIB chamber for micro-patterning. Figure 3a shows a SEM image of the flat MN after the micro-patterning with FIB, and it shows that the edges of the needle are slightly covered by residual wax. Consequently, the patterned MN was cleaned prior to any permeation experiments. 3D microscopy images as shown in Fig. 3b, c were also taken of the patterned MNs in order to obtain a depth profile of the patterned MN to ascertain if they were successfully patterned to the specification outlined. The 3D microscopy results showed that the pattern and the profile were consistent. Therefore, the SEM images produced at the time of ablation will be sufficient enough to visualise the tips of the well-defined FIB needle. The images of the tip were taken after they were cleaned. In the case of the microscopy images, it is shown that SEM produced a clearer image of the FIB structure than using 3D microscopy. However, the 3D microscopy can give an indication of the height of the patterned MN tips which was shown to be approximately 4 μm. Two views of the commercially available sharp microneedle patch which we have used in this work to pre-treat the porcine skin are shown in Fig. 3d, e.

### Skin permeation of BSA

The concentration of BSA in the receptor chamber was calculated over a period of 5 h (300 min) from the time when the donor compound of concentration 1000 μg/ml BSA was placed into the donor chamber. Full thickness of porcine skin was used as the membrane. Four parameter experiments were conducted: passive diffusion (no MN insertion), the use of well-defined patterns on the tip of flat MN to ablate the skin (FIB MN), fabricated short flat MN (ablation of the skin to act like sand paper) and the insertion of a sharp MN. A repeat of ten experiments was conducted and the average results presented. The results for the FIB fabricated patterned MNs showed a similar BSA release profile compared to flat MN, therefore indicating that the patterned MN showed little to no difference in drug permeability as that of flat MNs. The MN was placed onto the porcine skin for 3 min using the pneumatic pump. Passive diffusion was used as a control.

**Fig. 4:**
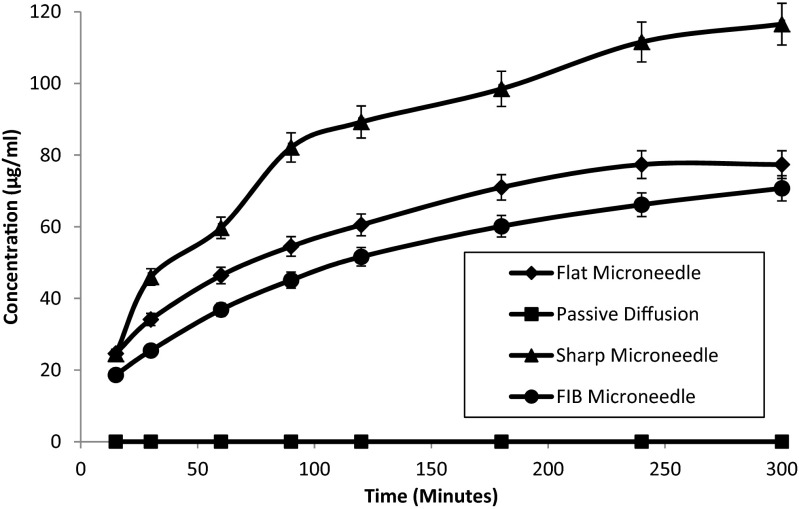
Concentration of bovine serum albumin (BSA) over a period of 300 min when a solution of BSA concentration 1000 μg/ml is applied onto full thickness porcine skin in vitro, with no microneedle (passive diffusion) and insertion of flat microneedle, FIB microneedle and a long sharp microneedle for a period of 3 min. All experiments have been done with ten repeats

It was observed (Fig. 4) that the concentration of BSA increased over time for all MN insertions but produced no concentration of BSA for passive diffusion. This is because the molecular size of BSA is too large to passively diffuse through the SC layer of the porcine skin. Similar results have been shown in the literature [22]. After 5 h, the concentration of BSA when a sharp MN was applied to porcine skin showed an approximate concentration of 120 μg/ml compared to no insertion of MN on porcine skin. Whereas after 5 h, the concentration of BSA when a flat MN 250 μm diameter, 250 μm length, was applied to porcine skin showed an approximate 80 μg/ml concentration difference compared to no insertion of MN on porcine skin. The patterned MN gave a 7-μg/ml less concentration compared to the flat MN. This shows a small drug release difference compared to the sharp MN, and therefore, it is observed that the patterned and flat MN gave similar drug release profiles.

The results for the insertion of flat and sharp MNs showed a similar trend with the concentration of BSA being approximately 40 μg/ml greater for sharp MNs than flat MNs over 5 h. There is a greater diffusion of BSA through the porcine skin when a MN is inserted into the porcine skin compared to no MN insertion. This is because the MN created channels in the skin which allow the passing of a larger concentration of BSA to diffuse through the full skin thickness. The channels that have been opened allow a greater diffusion environment allowing large molecules to permeate through the porcine skin. Flat MNs show a promising permeability of MN compared to sharp MNs, where by the skin is ablated rather than directed pierced. Similar results have been shown in the literature conducted by Wei-Ze et al. [5]. However, they showed that super-short MNs are capable of successfully delivering galanthamine (GAL) with a higher permeation than sharp MNs. From our results, it can be observed that the flat MNs give lower concentration profiles to the chosen sharp MNs. However, the concentration difference is not different enough to suggest that flat MN could not be used as an alternative to sharp MNs. This would infer that the flat MNs could be used as an alternative to conventional long sharp MNs and avoid the problems associated with pain from long MNs [23].

It has been shown in the literature by Han and Das [24] that the amount of drug release as a result of MN insertion is largely affected by the length of the MNs themselves. Therefore, the resultant drug release profile from insertion of the sharper MN compared to the insertion of flat and well-defined MNs seems to be consistent with the observations made by Han and Das [24]. The sharp MN is nearly six times the length of the other two MNs used. As there is a slight height difference in the MN length for the flat and well-defined MNs of approximately 4 μm, the question to ask is whether this is a significant height variation in MN length to deduce a significant difference in drug release profile. As Han and Das have illustrated the actual MN penetration depth in the skin is not the same as the length of the MN, we have assumed that this height difference is negligible in this case.

## Conclusion

It was shown that the delivery of BSA with fabricated flat microneedles (approximate concentration of 80 μg/ml) gave a similar drug release profile in comparison to well-defined FIB fabricated patterned MNs (approximate concentration of 70 μg/ml) after 5 h. The sharp MNs showed an increase of drug release in comparison to the flat MNs, but they are expected to be more painful when inserted into the skin. Passive diffusion gave no permeation data, as would be expected due to the large molecular size of the molecule. The results for the sand paper like MNs fabricated using FIB showed similar BSA release as the flat MNs (250 μm diameter, 250 μm length), therefore indicating that the sand paper MNs showed negligible difference in drug permeability as that of the flat MNs. The results show that using FIB as a technique to create a sandpaper effect to porate the skin is an effective tool.
